# Characterization of phosphorus-regulated miR399 and miR827 and their isomirs in barley under phosphorus-sufficient and phosphorus-deficient conditions

**DOI:** 10.1186/1471-2229-13-214

**Published:** 2013-12-13

**Authors:** Michael Hackenberg, Bu-Jun Shi, Perry Gustafson, Peter Langridge

**Affiliations:** 1 Computational Genomics and Bioinformatics Group, Genetics Department, University of Granada, 18071 Granada, Spain; 2 Australian Centre for Plant Functional Genomics, The University of Adelaide, Urrbrae, South Australia 5064, Australia; 3 USDA-ARS, 206 Curtis Hall, University of Missouri, Columbia, MO 65211-7020, USA

**Keywords:** Barley, Phosphorus, miR399, miR827, Isomir, Antisense, Targets, Differential expression

## Abstract

**Background:**

miR399 and miR827 are both involved in conserved phosphorus (P) deficiency signalling pathways. miR399 targets the *PHO2* gene encoding E2 enzyme that negatively regulates phosphate uptake and root-to-shoot allocation, while miR827 targets SPX-domain-containing genes that negatively regulate other P-responsive genes. However, the response of miR399 and miR827 to P conditions in barley has not been investigated.

**Results:**

In this study, we investigated the expression profiles of miR399 and miR827 in barley (*Hordeum vulagre* L.) under P-deficient and P-sufficient conditions. We identified 10 members of the miR399 family and one miR827 gene in barley, all of which were significantly up-regulated under deficient P. In addition, we found many isomirs of the miR399 family and miR827, most of which were also significantly up-regulated under deficient P. Several isomirs of miR399 members were found to be able to cleave their predicted targets *in vivo*. Surprisingly, a few small RNAs (sRNAs) derived from the single-stranded loops of the hairpin structures of MIR399b and MIR399e-1 were also found to be able to cleave their predicted targets *in vivo*. Many antisense sRNAs of miR399 and a few for miR827 were also detected, but they did not seem to be regulated by P. Intriguingly, the lowest expressed member, hvu-miR399k, had four-fold more antisense sRNAs than sense sRNAs, and furthermore under P sufficiency, the antisense sRNAs are more frequent than the sense sRNAs. We identified a potential regulatory network among miR399, its target *HvPHO2* and target mimics *HvIPS1* and *HvIPS2* in barley under P-deficient and P-sufficient conditions.

**Conclusions:**

Our data provide an important insight into the mechanistic regulation and function of miR399, miR827 and their isomirs in barley under different P conditions.

## Background

Phosphorus (P) is a major component of fundamental macromolecules in organisms and plays multiple roles in plants, especially in energy transfer, metabolic pathways and regulation of enzymatic reactions. However, P is the second most limiting element for plant growth after nitrogen. Typically, its availability in soil is too low to meet the requirements of plant growth. Therefore, many efforts have been made in the past to improve the ability of plants to acquire P from soils. These efforts have included the utilization of P transporters and, recently, microRNAs (miRNAs) [1-10]. miRNAs are a novel class of non-coding small RNAs (sRNAs) of approximately 22 nucleotides (nt) in length. They are generated from stem loop-forming miRNA precursors (pre-miRNAs), which are processed from long primary miRNAs (pri-miRNAs), transcribed from genomic DNA. In cytoplasm, only one strand (guide strand) of miRNA is loaded into the miRNA induced silencing complex (miRISC) and guides the Argonaut protein (AGO) in miRISC to recognise and cleave mRNA targets or to inhibit protein translation of the targets [11]. Another strand (passenger strand) of the miRNA is degraded via the degradation pathway. Despite their small size, miRNAs play an important role in the regulation of gene expression in various organisms. Compared with other classes of RNAs including mRNAs, miRNAs are more conserved across species.

With the advent of deep sequencing technology, multiple miRNA variants have been identified in various species, tissues and physiological conditions [12,13]. These miRNA variants are termed isomirs [14], and result either from imprecise or alternative cleavage by the RNaseIII enzymes Dicer (in plants) or Drosha (in animals) during miRNA biogenesis, or from post-transcriptional editing. Isomirs can be classified into three major types: length variations, nt variations along the miRNA and non-templated additions (NTAs) such as adenylation and uridylation at the 3′ end [15]. All three types of isomirs have been suggested to have a biological role [12,13]. However, it is not clear if the generation of isomirs is a process that is regulated according to environmental conditions. The capacity and specificity of isomirs to regulate their targets relative to their canonical miRNAs are also not well understood.

miR399 is the first miRNA demonstrated to have an ability to increase plant uptake of P in plants [8,16-20]. Its target has been identified as *UBC24*, which encodes a ubiquitin-conjugating E2 enzyme, also known as PHOSPHATE 2 (PHO2) [9]. In transgenic plants, increasing miR399 transcripts reduces UBC24 transcripts [21]. It has been shown that the 5′-untranslated region (UTR) of *UBC24*/*PHO2* contains multiple sequences complementary to miR399 [22]. Removal of these sequences stabilises the level of *UBC24*/*PHO2* transcripts under P deficiency [16], indicating that these sequences are likely to be targeted by miR399. The miR399 relationship with *UBC24*/*PHO2* in response to P starvation has been proposed to be conserved in angiosperms [20,23,24].

However, the miR399 activity in targeting *PHO2* is quenched by *IPS1* (Induced by Phosphate Starvation 1), a long non-coding RNA containing a sequence motif complementary to miR399 [25]. *IPS1* functions as a target mimic by using its motif to sequester miR399 from *PHO2*, thereby protecting *PHO2* from miR399-mediated cleavage. Because *IPS1* lacks a miRNA-mediated cleavage site, the interaction between *IPS1* and miR399 is stable. Interestingly, the expression of *IPS1* is suppressed by *PHO2 per se*, revealing autoregulation of miR399 and *PHO2*.

Another P-upregulated miRNA under P deficiency is miR827 [26-30]. miR827 targets *SPX-MSF* genes, dependent on P conditions. Under low P conditions it targets *SPX-MSF1* while under optimal P conditions it targets *SPX-MSF2*[28]. The *SPX-MFS* genes have been predicted to be implicated in phosphate (Pi) sensing or transport [28]. In Arabidopsis, miR827 is also shown to target the Nitrogen Limitation Adaptation (*NLA*) gene, thereby playing a pivotal role in regulating Pi homeostasis in a nitrate-dependent fashion [29].

Currently, little information is available on the relationship of miR827 or miR399 expression with P conditions in cereal crops. In this study, by analysing expression profiles of miR399 and miR827, we identified 9 novel members of the miR399 family, and many isomirs and antisense sRNAs for both miR399 and miR827. Importantly, we identified several functional isomirs of the miR399 family. Additionally, several small RNAs (sRNAs) derived from the single-stranded loops of the hairpin structures of two MIR399 genes were also shown to be functional by the detection of cleavage products of their predicted targets. Furthermore, we also identified a potential regulatory network among miR399, its target *HvPHO2* and target mimics *HvIPS1* and *HvIPS2* in barley under P-deficient and P-sufficient conditions. Our data provide an important insight into the mechanistic regulation and function of miR399 and miR827 in barley under different P conditions.

## Results

### Presence of miR399 and miR827 genes in the barley genome

To date, 159 miR399 genes (155 unique sequences) and 16 miR827 genes (16 unique sequences) have been identified from various species and deposited in miRBase (Release 19: August 2012). All the 159 mature miR399 members (39 unique sequences) and 16 mature miR827 members (9 unique sequences) originate from 3p arms. The deposited 5p arm sequences in miRBase are only 19 (18 unique sequences) for miR399 genes and 2 (2 unique sequences) for miR827 genes. There is a dichotomy between the numbers of mature miRNAs and unique sequences in the database. This can be explained by the very high level of conservation between miRNAs of different species. Although many miR399 and miR827 members/genes have been identified in various plant species, currently only one miR399 sequence, but no miR827 sequence, from barley is deposited in miRBase. Given this scenario, we took advantage of our previously obtained sRNA datasets from shoots of P-deficient and P-sufficient barley [30] to identify additional miR399 and miR827 genes in the barley genome. We first aligned all the sRNA sequencing reads to miRBase without mismatch, identifying seven miR399-like sRNA sequences with a total of 644 reads in P-deficient barley and five miR399-like sequences with a total of 62 reads in P-sufficient barley (Additional file 1: Table S1). All of the five miR399-like sequences from P-sufficient barley were detected and up-regulated in the P-deficient barley dataset. When up to 2 nt mismatches were allowed, 10 additional miR399-like sequences were identified in P-deficient barley (red sequences in Additional file 1: Table S1), but no additional miR399-like sequences were found in P-sufficient barley. One of the miR399-like sequences was aligned with miR399d in antisense (Additional file 1: Table S1). Compared to miR399, only two miR827-like sequences, corresponding to osa-miR827a and ghr-miR827a, respectively, were perfectly matched in miRBase from the barley sRNA datasets (Additional file 1: Table S1). However, the homolog of ghr-miR827a does not map to the barley genome (Additional file 1: Table S1). When one nt mismatch was allowed, 50 and 26 miR827-like sequences were further identified in P-deficient and P-sufficient barley, respectively (Additional file 1: Table S1). Of these, 24 were only detected in P-deficient barley while two were specific to P-sufficient barley (Additional file 1: Table S1). Twenty of the miR827-like sequences common to both datasets were up-regulated in P-deficient barley, while three were down-regulated (Additional file 1: Table S1).

The detection of miRNA genes by simply mapping the sRNA sequencing reads to miRBase may generate several errors: (i) some miRNA ‘genes’ might not have a stem-loop structure or low mean free energy, because their pre-miRNA sequences have not been assessed, and hence they may not be true miRNA sequences; (ii) some of the mapped reads might be isomiRs (sequence variants of the canonical mature miRNA [13] and hence not distinct miRNAs); and (iii) some reads may have been erroneously generated from sequencing errors. In line with these points, we found that nine miR399-like sequences and all of the miR827-like sequences (except for the homolog of osa-miR827a) do not perfectly map to the barley genome (Additional file 1: Table S1). We thus consider these sequences to be false positive detections. Some of these sequences may be isomirs, as some nt variations occur at the 5′ ends or internal sequences, which are unlikely to be generated by sequencing errors.

In order to improve the detection of miR399 and miR827 genes, we used a prediction based on the miRanalyzer algorithm [31], together with the newly available barley genome assemblies [32]. In total, we found ten miR399 genes and one miR827 gene. The ten miR399 genes are hvu-miR399-1, hvu-miR399-2, hvu-miR399-3 and hvu-miR399-4, miR399b, miR399c, miR399d, miR399e-1, miR399e-2 and miR399k (Additional file 2: Table S2). The first four miR399 genes give rise to the same mature miRNAs and hence they are given the same name, but with a unique suffix of an identifier number. Of the identified miR399 genes, only one (hvu-miR3993p) is currently annotated in miRBase, but the corresponding 5p arm is not annotated in miRBase. The other nine miR399 genes are novel (Additional file 2: Table S2). miR399-2 and miR399-3 not only have the same miR399-3p sequence but also have the same miR399-5p sequence, which was covered by five reads in our experimental data from P-deficient barley. The 5p sequence of miR399-4 was not detected in our datasets, but the theoretical sequence only differs in one position from the miR399-2-5p sequence. It is very likely that the hvu-miR399-3p sequence in miRBase is derived from hvu-miR399-2, as some reads mapped uniquely to this sequence and not to the other three miR399 genes. Furthermore, the annotated miR399 gene in miRBase does not show a canonical biogenesis, allowing us to rule out that the mature guide strand was obtained from this gene. This does not necessarily mean that the miRBase MIR399 gene is a false positive, but only that in barley shoots it seems not to be processed accordingly to a miRNA. For miR399e, which includes two genes, miR399e-1 and miR399e-2, we found both genes give rise to the same miR399e-3p sequence but have different miR399e-5p sequences. The remaining miR399 genes have both 3p and 5p arm sequences detected from the sRNA datasets. All of the pre-miR399s form stem-loop structures, with mature miRNAs located in the stems (Additional file 3: Figure S1).

**Figure 1:**
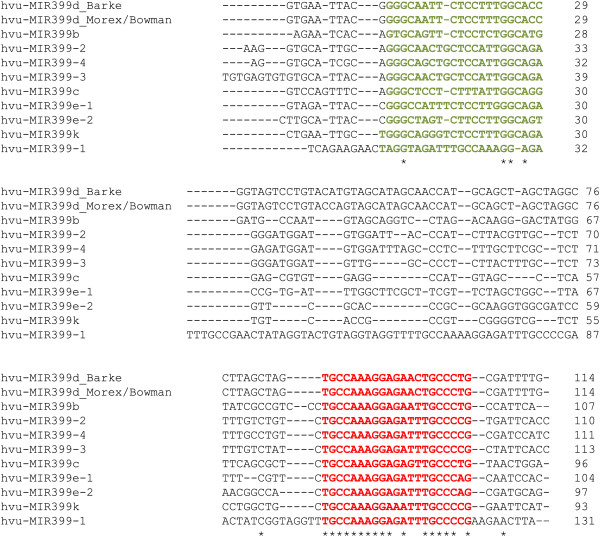
**Sequence alignments of barley pre-miR399 genes.** Sequences highlighted in red are hvu-miR399-3p sequences, while those highlighted in green are hvu-miR399-5p sequences. Conserved nucleotides are indicated by “*”. For hvu-miR399d, suffixes Morex, Barke and Bowman indicate the genomic sequences of the barley cultivars from which pre-miR399 sequences were derived.

Alignment of miR399 3p sequences (red sequences in Figure 1) revealed high homology. Only three nt vary among the ten miR399-3p sequences. By contrast, the miR399 5p sequences (green sequences in Figure 1) only share three conserved nt. If the 5p sequence of hvu-miR399-1 is excluded, the conserved number of nt increases to seven. The overall sequence identity among the ten miR399 genes is about 35%. The lowest sequence identity is between hvu-MIR399-1 and hvu-MIR399c (39%), while the highest identity is between hvu-MIR399d from barley cv. Barke and hvu-MIR399d from cultivars Morex or Bowman (98%). By contrast, miR399 gene sequences in miRBase are less conserved, because they originate from different plant species (Additional file 4: Table S3).

By detecting novel miR399 genes at a genomic level, we could resolve that the perfect match of the read ‘TGCCAAAGGAGAATTGCCC’ to the miRBase sequence of tae-miR399 (see Additional file 1: Table S1) is a false positive, as this read mapped in the barley genome to the same location as miR399b, showing that it is a 3′ truncated isomiR of miR399b and not a novel miR399 gene homologous to tae-miR399. Examples such as this enforce the need for a genome sequence to reliably detect novel miRNAs. While many false positives detected using the miRBase mapping approach have low read counts, setting a stricter threshold on the read counts could result in bona-fide miRNAs being missed. For example, in our barley datasets, while miR399k gave low read counts, both arms were perfectly covered by sequencing reads.

Only one miR827 gene was found in the barley genome, whose 3p and 5p arms were both represented by reads (Additional file 2: Table S2). Surprisingly, from this gene other 3p and 5p arm sequences of miR827 were also detected by reads (Additional file 2: Table S2).

### Genomic location of barley miR399 and miR827 genes

Currently, three barley genome assemblies from varieties Morex, Bowman and Barke are available [33]. All the miR399 gene sequences, except for miR399-4, could be found in each of the three barley genome assemblies. miR399-4 is only found in the Morex and Bowman genome assemblies, but not in the Barke assembly (Additional file 2: Table S2). Similarly, the miR827 gene is only found in the Morex and Barke genome assemblies, but not in the Bowman assembly (Additional file 2: Table S2). The chromosomal locations of all miR399 and miR827 genes were determined from the barley assemblies (Additional file 2: Table S2), and were confirmed by Southern blot hybridization with barley-wheat addition lines (data not shown). The miR399 genes are distributed in the long arms of chromosomes 1H, 2H, 3H and 7H, of which 7HL contains five miR399 genes, while the others each contain two miR399 genes (Additional file 2: Table S2). The hvu-MIR399b and hvu-MIR399e-1 genes formed a cluster, as did hvu-MIR399-3 and hvu-MIR399-4 (Additional file 2: Table S2). The distance between the two genes in each cluster is around 2000 nt (Additional file 2: Table S2). The orientation of hvu-MIR399-3 is the same as that of hvu-MIR399-4, while the orientation of MIR399b is the same as that of hvu-MIR399e-1 (Additional file 2: Table S2). Consistently, miR399 members in the rice genome were also found to form clusters [34].

Further location analysis showed that hvu-MIR399b is localized within an intron, while hvu-MIR399e-1 is localized within an exon of a protein-encoding gene confirmed by RT-PCR. hvu-MIR399c, hvu-MIR399d and hvu-MIR399k were also localized within introns. Intriguingly, hvu-MIR399-1, hvu-MIR399-2, hvu-MIR399-3, hvu-MIR399-4, hvu-MIR399e-1 and hvu-MIR399k each have one or two homologous sequences either in a cluster form or in different locations. It is unclear whether these loci are functional. hvu-MIR827 locates to chromosome 2HL (Additional file 2: Table S2), within an intronic region of a gene.

### Expression profile of miR399 and miR827 genes under different phosphorus conditions

To compare the obtained read counts (number of times a given RNA molecule was sequenced) between the sequence datasets obtained from P-sufficient and P-deficient barley, we used the RPM (Reads Per Million) expression value, calculating the number of reads mapped to a given element out of each 1,000,000 input reads. This measure is independent of the total number of input reads and can be used for an unbiased comparison between different samples. We found that MIR827 was more highly expressed than any of the MIR399 genes (Additional file 5: Table S4). In P-deficient shoots, we detected 9559 (RPM = 3341) sequence reads corresponding to the guide strand (3p) while under P-sufficiency only 1203 (RPM = 592) reads were detected (Additional file 5: Table S4). This difference corresponds to a fold-change of nearly 6. The RPM expression value shows that under P deficiency, miR827-3p made up nearly 0.33% of all mapped input reads.

Among MIR399 genes, miR399k-3p was the lowest expressed (RPM = 1.4). The other MIR399 genes varied within a very small range from 50.7 (miR399d-3p) to 93.3 (miR399e-1-3p). Although the expression values of the guide strand were similar among the different MIR399 genes, the fold-changes between P-deficient and P-sufficient samples varied significantly. For example, miR399e-3p was expressed over 90-fold more highly under P deficiency, whereas the increase in miR399c-3p expression under P deficiency was only 2.3-fold. The most strikingly P-regulated MIR399 gene was miR399d, for which we could not find a single read in the P-sufficient dataset. By contrast, we identified 145 reads belonging to the guide strand of this miRNA in the P-deficient dataset. It is not known how miR399d is regulated under P conditions, but there is likely to be a P-controlled switch for expression of this miRNA under certain P conditions.

As expected, we generally observed high ratios between the read counts of the guide strand and the passenger strand for most MIR827 and MIR399 genes (see ‘Arm ratio 3p/5p’ in Additional file 5: Table S4). For example, for MIR827 we found nearly 41 times more reads from the 3p strand while for MIR399-1 and MIR399d we found the ratio to be around 29 (Additional file 5: Table S4). Those high ratios strongly suggest that the passenger strand is degraded while the guide strand is functional. However, there were three notable exceptions in the data in Additional file 5: Table S4: [1] although at an overall low read count, the read count of the passenger strand (5p) of MIR399k was five times higher than that of the guide strand (3p) (20 reads vs. 4 reads); [2] there were almost the same number of reads for each of the 3p and 5p arms of MIR399b (150 reads from the 5p arm vs. 177 reads from the 3p arm); and [3] for MIR399c the reads from the guide strand were only slightly higher (1.9 times) than those from the passenger strand. Additionally, a modified read from miR399c-5p was observed, which might have a functional miRNA/mRNA interaction.

Intriguingly, for both MIR399c and MIR399b, for which we observed low 3p/5p arm ratios, we found that the 5p passenger strand is differentially expressed more strongly than the guide strand (Additional file 5: Table S4) due to P conditions. The 5p arm of MIR399b shows a 106.7-fold increase in expression under P deficiency, while the guide strand (3p) is only up-regulated by 9.7 folds (Additional file 5: Table S4). Similarly but less pronouncedly, for miR399c-5p we observed a 4.7-fold increase in expression in the P-deficient barley, while for miR399c-3p there was only a 2.4-fold increase (Additional file 5: Table S4). This stronger differential expression of the passenger strand might suggest the presence of an alternative biogenesis pathway in which the passenger strand is incorporated (more frequently) into the miRISC complex. The detected target cleavage product for miR399c-5p could support this possibility.

**Figure 2:**
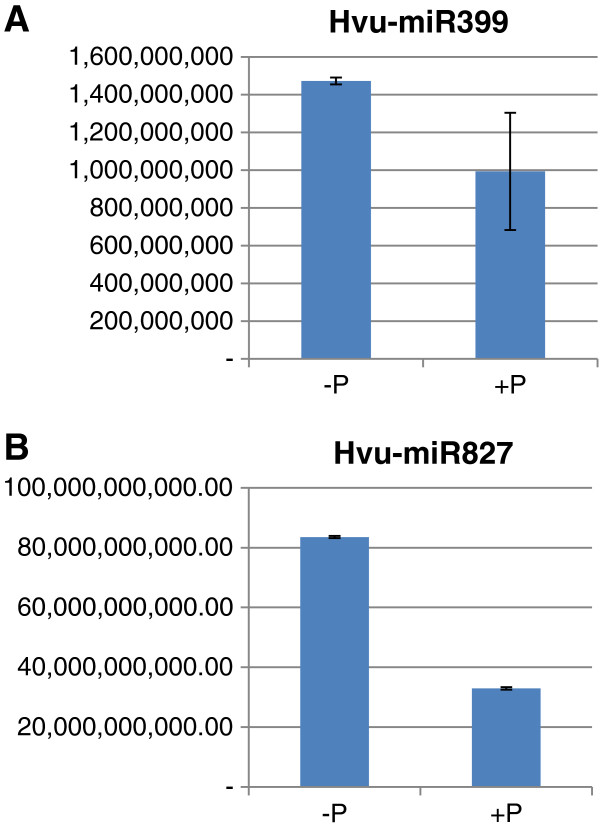
**qRT-PCR analysis of the expression of mature miR399 and miR827 in P-deficient (−P) and P-sufficient (+P) barley shoots.** Mature miRNA sequences were used as forward primers while an oligo(dT) sequence was used as a universal reverse primer for both miR399 **(A)** and miR827 **(B)**. Please note that the expression level of miR827 is much higher than that of miR399.

**Figure 3:**
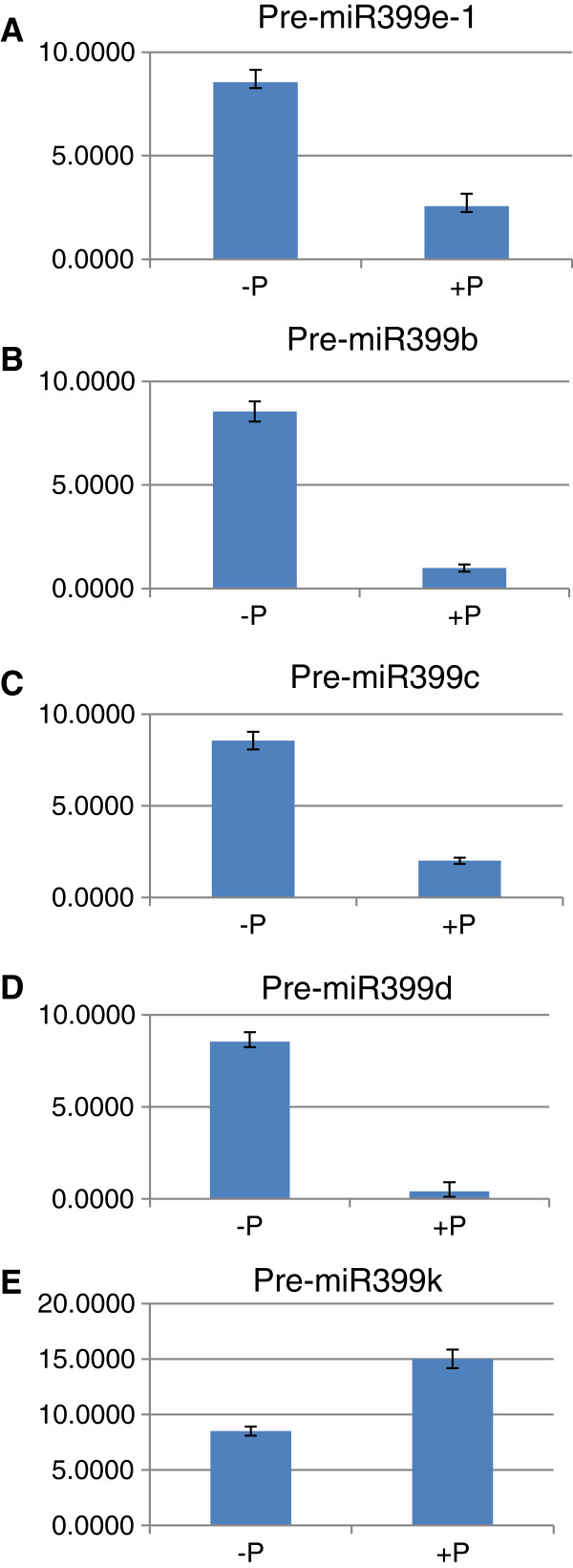
**qRT-PCR analysis of the expression of pre-miR399 and pre-miR827 genes in P-deficient (−P) and P-sufficient (+P) barley shoots.** Specific forward and reverse primers were designed from the 5′ terminal sequences and 3′ terminal sequences, respectively, of pre-miR399 and pre-miR827 members. **A**: Pre-miR399e-1; **B**: Pre-miR399b; **C**: Pre-miR399c; **D**: Pre-miR399d; **E**: Pre-miR399k.

To better estimate the expression levels of miR399 and miR827 under different P conditions, quantitative real-time PCR (qRT-PCR) was performed. We firstly used a forward primer specific to mature hvu-miR399 or hvu-miR827 sequences, and a universal reverse primer. The expression level of hvu-miR399 was 1.5-fold higher under P-deficient conditions than under P-sufficient conditions (Figure 2A), which was consistent with the read abundance of this miRNA in the P-deficient and P-sufficient barley sequencing datasets. This suggests that sRNA sequencing data can be used for assessing the expression levels of sRNAs. Similar to miR399, the expression level of miR827 was also 2.5-fold higher in P-deficient barley than in P-sufficient barley (Figure 2B), suggesting that both miR399 and miR827 may function in P homeostasis in barley. The expression level of miR827 was 50-fold higher than that of miR399 under P-deficient conditions (Figure 2A and B). Because the initial primers used for qRT-PCR could not distinguish between different members of the miR399 family, we designed specific forward and reverse primers according to the pre-miR399 sequences (Additional file 6: Table S5). qRT-PCR analysis showed that four pre-miR399 genes were expressed at much higher levels in P-deficient barley than in P-sufficient barley (Figure 3), which is consistent with previous studies [12]. However, pre-miR399k was expressed at a lower level in P-deficient barley than in P-sufficient barley (Figure 3E). All the other pre-miR399s were expressed at very low levels in both P-deficient and P-sufficient barley, below the detection limits of qRT-PCR (data not shown). These results, combined with the read abundance of mature miR399s, reveal the existence of different expression between the mature miRNAs and the pre-miRNAs in the cells. The expression profile of pre-miR827 was found to be similar to its mature miRNA, which was about 2.5-fold higher in P-deficient barley than in P-sufficient barley (data not shown). Taken together, these results indicate that the expression of miR399 members and miR827 are clearly affected by P conditions in barley. However, how they are affected by P and whether the resultant differential expression is involved in different functions in barley remain unclear.

### Profile of miR399 and miR827 isomirs and alternative biogenesis pathways

Different putatively functional sRNAs can be generated from the same pre-miRNA sequence [35]. A typical example is isomirs, which have been shown to be functional in plant species [36]. Recently, isomirs became the focus of miRNA research and most miRNA variants might have the capability to change the target gene repertoire of the canonical sequence. Analysis of isomirs of miR399 and miR827 showed that both adenylated and uridylated reads were present for all guide strand mature sequences under P deficiency (Additional file 7: Table S6). Under P-sufficient conditions, we only found uridylated or adenylated reads for MIR827, but not for MIR399-1. Interestingly, we found that the strongest modified mature sequence under P deficiency is hvu-miR399c-5p, which has nearly 10% of uridylated reads (Additional file 7: Table S6). Furthermore, MIR399c is the least differentially expressed miRNA, perhaps due to the destabilizing potential of uridylation [37]. miR399c-3p, miR399-3p and miR399k-3p did not show adenylation and uridylation at their 3′ ends (Additional file 7: Table S6).

A previous study showed that the most frequent length variants of miRNAs are 1 nt shorter or longer than the canonical version at their 3′ and 5′ ends [38]. However, we found that the most frequent length variant in MIR399-1 is one truncated by 3 nt at the 5′ end, which almost makes up 10% of the total variants, for example, those in miR399c-3p (Additional file 7: Table S6). These variants have potentially different targets compared to the canonical sequence.

Apart from the generation of isomirs, we also analysed if there might be any other differences in the biogenesis of miRNAs under P sufficiency and P deficiency. We observed that MIR399-1 generates a number of sRNAs under P deficiency which are absent under P sufficiency (Additional file 3: Figure S2). The alignment pattern shown in Additional file 3: Figure S2 indicates that at least four different and unique sRNAs are generated from the MIR399-1 pre-cursor sequence. However, we didn’t find targets for these sRNAs in our degradome library, so currently we cannot confirm that the generation of these fragments has function.

Another interesting observation is that MIR827 generates two, overlapping bona fide miRNAs (Additional file 2: Table S2 and Additional file 3: Figure S3). Apart from the canonical sequence, we found a second pair of mature/mature* miRNA sequences that show a perfect 2 nt 3′ overhang as expected from a canonical DCL processing. In plants, it was previously observed that a single pre-miRNA sequence can give rise to several different sRNA molecules. However, as far as we know this is the first time that two different, but overlapping mature miRNAs have been detected. This suggests that within barley shoots, the MIR827 precursor is processed in two different ways: i) to generate the canonical form which shows dramatic differences between P deficiency and P sufficiency in its frequency; and ii) the alternative form that overlaps with the canonical and which does not show differential frequency between P deficiency (RPM: 2.8) and P sufficiency (RPM: 3.1). Only the canonical biogenesis pathway appeared to be regulated by P.

Surprisingly, we found a few sequence reads from the loops of the hairpin structures of MIR399b and MIR399e-1, although the read length of these was short, ranging from 16 nt to 19 nt only (Additional file 3: Figure S4). Loop-derived sRNAs have been found previously in *Drosophila*[39-41] and have been shown to be functional [42,43], suggesting that the reads derived from the single-stranded loops of MIR399e-1 and MIR399b in barley may not be randomly generated and may be stable and active in cells. This would in turn suggest that they contribute to target specificity, as their sequences are different from the canonical miRNAs from the same pre-miRNAs.

### Targets of miR399 and miR827 and their isomirs in barley

Targets of miR399 and miR827 were first predicted using psRNAtarget. A total of 46 targets for the miR399 members and 12 targets for miR827 were predicted (Additional file 8: Table S7). Some targets have functional annotations, while others do not. A number of targets contain multiple target sites, and are targeted by either the same miRNA or different miRNAs. For example, the gene encoding DNA primase (AV835204) has two predicted sites specifically targeted by hvu-miR399g, while *HvPHO2* has five predicted target sites, targeted by different miR399 members (Additional file 8: Table S7).

Predicted targets of miR399 and miR827 were validated using a barley degradome library constructed by RLM-5′-RACE (RNA ligase-mediated rapid amplification of 5′ cDNA ends) technology. The advantage of using a degradome library is that the cleavage sites of miR399 or miR827 targets can be identified without requiring a *priori* miRNA target prediction. In total, 160 targets of miR399 and 22 targets of miR827 were detected in the library (Additional file 9: Table S8), some of which corresponded to target identities that were predicted bioinformatically, while others had not been predicted. The detected targets of miR399 and miR827 indicate that both miRNAs have diverse and complicated function. It is to note that the targets listed in Additional file 9: Table S8 also include those of putative miR399 and miR827 genes that were not identified in our barley sRNA datasets but were annotated in miRBase.

**Figure 4:**
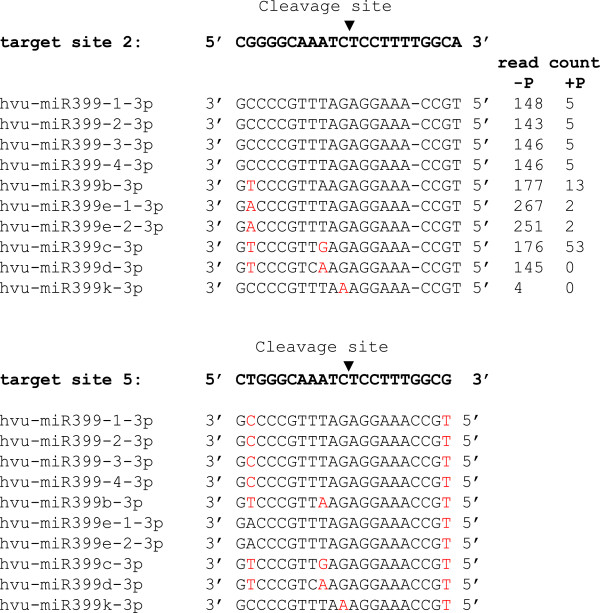
**Alignment of miR399 members to two predicted cleavage sites **[2]**,**[5]**on *HvPHO2*.** Mismatched nucleotides (nt) are indicated in red, while a missing nt is represented by “-“.

Consistent with previous studies, many cleavage sites occurred at positions 10–11 relative to the 5′ end of the miRNA (Additional file 9: Table S8). However, cleavage sites occurring at other positions within the targeted site were also observed (Additional file 9: Table S8). Intriguingly, although *HvPHO2* targeted by miR399 contains five predicted miR399-interacting sites in the 5′ untranslated region [44], only two of these (interacting sites 2 and 5) were found to be cleaved, and occurred at positions 10–11 relative to the 5′ end of the miRNA (Figure 4). By contrast, in *Arabidopsis* four of the five miR399-interacting sites [2-5] in the *PHO2* gene were reported to be cleaved [21,45]. Why the other three miR399-interacting sites could not be cleaved by miR399 in barley is unknown, but likely linked to the expression level of the miR399 member(s) that specifically cleave the site, and this may be tissue- or condition-dependent. With the exception of miR399d, all miR399 members cleaved both interacting sites 2 and 5 (miR399d cleaved interacting site 2 only). Furthermore, all miR399 members cleaved the *HvPHO2* target at an equal efficiency as judged by the fragment frequency.

**Figure 5:**
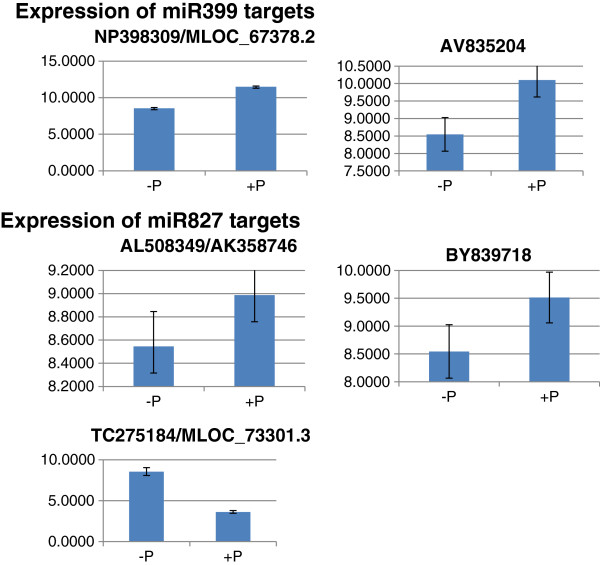
**qRT-PCR analysis of the expression of predicted targets of miR399 and miR827 in P-deficient (−P) and P-sufficient (+P) barley shoots.** Primer pairs flank cleavage sites of the predicted targets defined by the bioinformatics prediction and degradome library. NP398309 (MLOC_67378.2), AV835204, AL508349 (AK358746), BY839718 and TC275184 (MLOC_73301.3) are accession numbers or gene names from the MIPS Barley database (in bracket) of the predicted targets of miR399 or miR827, encoding NBS-LRR-like protein, DNA primase, proline rich protein, an unknown protein and cytochrome P450-like protein, respectively.

To further confirm the targets of miR399 and miR827, some of the targets detected in the degradome library for miR399 and miR827 were selected for qRT-PCR to determine their expression levels. It is expected that a given miRNA and its target would show an inverse relationship in expression level. Indeed, qRT-PCR analysis showed that when the levels of miR399 or miR827 increased under P deficiency, the levels of most of the selected target transcripts decreased (Figure 5). However, one target (Accession No. TC275184) of miR827, which encodes cytochrome P450-like TBP (TATA box binding protein) and plays important endogenous and exogenous roles in the oxidative metabolism [46], did not reduce its expression level in P-deficient barley where the level of miR827 increased (Figure 5). On the other hand, its cleavage product was detected in the degradome library and many P450-derived sRNAs were detected in the sRNA datasets. Therefore, it is inferred that other unknown factor(s) may be involved in the expression of miR827, which is irrelevant to the regulation of the target. It is worth mentioning here that this target is named as MLOC_73301.3 and annotated as Aquaporin 1 in the MIPS Barley database [47]. In order not to be tangled, all the genes described below would be given both Accession No and gene name from the MIPS Barley database. If the genes are not found in the MIPS Barley database, then they would be given only Accession No.

Targets of isomirs of miR399 and miR827 were also directly examined using the barley degradome library. We found several targets for isomirs derived from MIR399-4, MIR399c, MIR399b and MIR399d genes but not for those derived from other miR399 genes or from miR827 (Additional file 10: Table S9). These functional isomirs include mono-adenylated reads from miR399c-5p (Additional file 10: Table S9). In addition, we found that one target (AV835204) was cleaved by isomirs derived from three miR399 genes at two different positions (Additional file 10: Table S9). Furthermore, this target can also be cleaved by their canonical miRNAs.

As described above, several sequence reads were identified from the loops of the hairpin structures of MIR399b and MIR399e-1 (Additional file 3: Figure S4), and were implied to be functional. Accordingly, in the barley degradome library we found that these reads cleaved their predicted targets (Additional file 11: Table S10).

### Antisense sequences of miR399 and miR827 in P-deficient and P-sufficient barley

The detection of both arm sequences of pre-miR399 and pre-miR827 in P-deficient and P-sufficient barley prompted us to investigate antisense sequences of miR399 and miR827. Surprisingly, every MIR399 or MIR827 was found to have an antisense sequence (Additional file 12: Table S11). The antisense sequences of hvu-MIR399d in P-deficient barley were even more abundant than their sense sequences (Additional file 12: Table S11). In P-sufficient barley, with the exception of hvu-MIR399c and hvu-MIR827, all of the miR399 antisense sequences were more abundant than their sense sequences (Additional file 12: Table S11). However, there were no significant differences in antisense read abundance for any of the hvu-MIR399 genes or the hvu-MIR827 gene, between P-deficient and P-sufficient barley (Additional file: 12: Table S11). By contrast and as described above, significant differences were observed for sense read abundance for all of the hvu-MIR399 genes and for the hvu-MIR827 gene between P-deficient and P-sufficient barley (Additional file 12: Table S11). The sense reads of hvu-MIR399d and hvu-MIR399k were not present in the P-sufficient barley dataset (Additional file 12: Table S11), suggesting that these two miRNAs are tightly regulated by P. Intriguingly, while MIR399k has no sense read in P-sufficient barley, its antisense reads are the second most abundant in this dataset (Additional file 12: Table S11). Perhaps the expression of sense RNA of MIR399k is sequestered by its antisense RNA under P sufficient conditions.

The antisense sequences of miR827 were not abundant in either the P-deficient or P-sufficient barley datasets (Additional file 12: Table S11). However, the ratio of sense to antisense miR827 was significantly different between P-deficient and P-sufficient barley. In P-deficient barley, this ratio was 179, while in P-sufficient barley it was only 13, which is a more than 13-fold difference between P-deficient and P-sufficient barley. Whether the high level of expression of miR827 in P-deficient barley results from a lower number of antisense sequences under P deficiency relative to P sufficiency is an interesting question to speculate upon. Some of the antisense reads identified for miR399 and miR827 may function as mimics to protect the targets of these miRNAs from cleavage. This could explain why some of the predicted targets of miR399 and miR827 were not identified in the barley degradome library (Additional file 9: Table S8). One miR399 mimic target has previously been identified in barley [44].

### Determination of miR399 cleaving HvPHO2

The target gene *HvPHO2* of miR399 contains five predicted miR399 target sites. By examining the degradome library, we confirmed that interacting sites 2 and 5 in *HvPHO*2 were cleaved by miR399. However, it was not known which of the ten miR399 members cleaved these sites. To determine this, we firstly aligned all miR399 sequences with interacting sites 2 and 5 in *HvPHO2*. We found that hvu-miR399-3p (including hvu-miR399-1-3p, hvu-miR399-2-3p, hvu-miR399-3-3p and hvu-miR399-4-3p) has a single nt deletion relative to target site 2, and two mismatches to target site 5 (Figure 4). By contrast, all the other miR399 members have a single nt deletion and one mismatch to target site 2, and 1–3 mismatches to target site 5 (Figure 4). hvu-miR399k contained a mismatch at position 11 relative to the 5′ end of the miRNA, to both target sites 2 and 5 (Figure 4). Next, we examined the abundance of the miR399 members in the sRNA datasets. As described, all of miR399s had similar read abundance under P deficiency except for hvu-miR399k, which was much less abundant (Figure 4). Taking these results together, we inferred that target site 2 would most likely be cleaved by hvu-miR399-3p (hvu-miR399-1-3p, hvu-miR399-2-3p, hvu-miR399-3-3p and hvu-miR399-4-3p inclusive), followed by hvu-miR399b-3p and hvu-miR399e-3p (miR399e-1-3p and hvu-miR399e-2-3p inclusive), while target site 5 would most likely be cleaved by hvu-miR399b-3p and hvu-miR399e-3p (miR399e-1-3p and hvu-miR399e-2-3p inclusive). hvu-miR399k may not be able to cleave the target due to its low expression level and the mismatch at position 11, which is a pre-requisite for this position to be cleaved. The role of this miRNA in barley is unclear.

Base-pairing of the other three target sites in *HvPHO2* with the miR399 genes was also analysed. None of the miR399 genes had less than 3 mismatches to target sites 3 and 4, thereby making these two sites the most diverse (Additional file 3: Figure S5). Furthermore, a mismatch at target site 4 occurs within the miRNA seed sequence important for recognising the target sequence, presumably making it difficult for this target site to be cleaved by miR399. In *Arabidopsis*, no mismatch occurs within the miRNA seed sequence to any of the five predicted target sites, and hence most of the target sites are cleaved [45]. Unexpectedly, although target site 1 in *HvPHO2* was perfectly matched to the abundant hvu-miR399-3p (hvu-miR399-1-3p, hvu-miR399-2-3p, hvu-miR399-3-3p and hvu-miR399-4-3p inclusive) (Additional file 3: Figure S5), its cleavage product by these or other miR399 genes was not found in the degradome library, suggesting that additional factor(s) may control the selection of miRNA cleavage sites.

### Correlation of miR399, its PHO2 target and IPS target mimics in P-deficient barley

**Figure 6:**
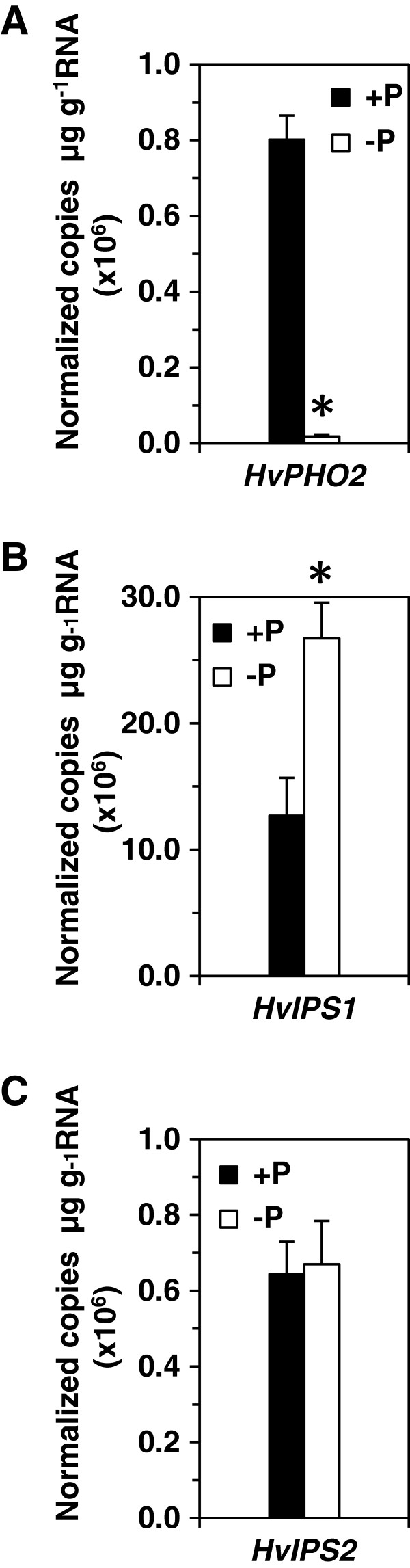
**qRT-PCR analysis of the expression of *HvPHO2, HvIPS1 *and *HvIPS2 *in P-deficient (−P) and P-sufficient (+P) shoots of barley (cv. Pallas).** The primers and conditions for the qRT-PCR were as previously described (Huang et al., 2011). **A**: qRT-PCR of HvPHO2; **B**: qRT-PCR of HvIPS1; **C**: qRT-PCR of HvIPS2. * means p < 0.003 versus the expression of the same gene under sufficient P.

Previous studies showed that P deficiency-induced *IPS* genes are target mimics of miR399 and can protect the targets of miR399, such as *PHO2*, from miR399-mediated cleavage [25]. We also showed that under P deficiency in barley (cv. Pallas) roots, where *HvIPS1* and *HvIPS2* were expressed at high levels [44], increased miR399 did not reduce the transcript level of *HvPHO2*, a *PHO2* ortholog in barley [44]. To see whether this is the same case in barley shoots, we examined the expression levels of these three genes (*HvPHO2, HvIPS1* and *HvIPS2*) under the same P conditions as for barley roots [44]. qRT-PCR showed that *HvPHO2* was expressed at higher levels under P-sufficient conditions than under P-deficient conditions (Figure 6), consistent with observations in *Arabidopsis*[8,9,16], but not in rice [48] or in barley roots [44]. By contrast, *HvIPS1* was expressed at higher levels under P-deficient conditions than under P-sufficient condition (Figure 6). However, *HvIPS2* expression did not differ between the two P conditions (Figure 6).

To further explore the correlation among hvu-miR399, *HvIPS1, HvIPS2* and *HvPHO2*, we aligned all the reads from the P-deficient and P-sufficient barley datasets with *HvIPS1, HvIPS2* and *HvPHO2* transcript sequences in both sense and antisense directions. We found no reads derived from *HvIPS1* or *HvPHO2* transcripts, but several reads derived from *HvIPS2*, in P-sufficient barley (Additional file 3: Figure S5). However, none of these reads were located in the miR399-interacting portion of the transcript sequence (Additional file 3: Figure S5). By contrast, *HvIPS1, HvIPS2* and *HvPHO2* transcript-derived reads were identified in the P-deficient barley dataset, although the read number for each transcript was limited (Additional file 3: Figure S5). In contrast to the reads derived from *HvIPS1* or *HvIPS2* transcripts, which were located outside the miR399-interacting sequence, the reads derived from *HvPHO2* were mostly located at miR399 target sites (Additional file 3: Figure S5). We also found a number of reads derived from the antisense strands of *HvIPS1* and *HvIPS2* transcripts, all of which were only found in the P-deficient barley dataset (Additional file 3: Figure S5). In addition, these reads were all located outside the miR399-interacting portions of the sequences (Additional file 3: Figure S5). Reads derived from the antisense strand of the *HvPHO2* transcript were also detected, more abundantly in P-deficient barley than in P-sufficient barley (Additional file 3: Figure S5). All of these reads were located at the target sites of miR399 (Additional file 3: Figure S5). These findings are important because they imply a miRNA regulation network that is more complex than expected. However, more work is required to gain an understanding of how these genes and sequences are coordinated, and regulated by plant P status.

## Discussion

We identified ten miR399 genes in barley. In rice nine miR399 loci exist [22,49], while in dicot Arabidopsis and soybean, six and five miR399 loci are present, respectively [22,50]. All of these miR399 genes are up-regulated under deficient P, but generally expressed at low levels in these plants. Numerous previous studies have shown that gene expression is controlled by upstream elements such as the promoter [51-54]. However, when we analysed upstream sequences of miR399 genes in plants, we could not find high sequence similarity for these upstream sequences. This suggests that the control of the expression of miR399 genes is independent. In support of this hypothesis, we found that different miR399 genes in barley are differentially expressed under the same conditions. This in turn suggests that other factors are involved, which may control the activity of upstream elements of miR399 genes. Such factors may either be common or unique to each miR399 gene, but associated with plant species, tissue specificity, developmental stage and/or environmental conditions.

In addition to the identified miR399 and miR827 genes, we also identified many isomirs of miR399 and miR827 in barley. Based on previous studies, these isomirs may not be attributable to sequencing errors or alignment artefacts [55-57], but are likely generated from the imprecise cleavage of Dicer or from post-transcriptional editing. Corresponding to this hypothesis, we found that the ends of most of the identified isomirs of miR399 and miR827 fit the type cleavage product from Dicer. However, some isomirs of miR399 or miR827 may be generated from different pathways, because their lengths vary significantly relative to their canonical miRNAs [58]. The specific origin of isomirs of miR399 or miR827 remains unclear. Intriguingly, we found NTAs at the 3′ ends of some isomirs of miR399 and miR827, and that these additions do not favour specific nt. A similar situation has been observed in animals, in which any nt can be added at the 3′ end of a miRNA [58-60]. However, uridylation (U) and adenylation (A) are relatively dominant NTAs. In animal miRNA isomirs, a U at the 3′ ends was proposed to help, while an A at the 3′ ends was anticipated to hinder, association with the miRISC [37]. However, we found that miR399 isomirs with an A addition also cleave their predicted targets *in vivo*. This suggests that the A addition differs little from the U addition in the ability to incorporate into the miRISC and permit function. In contrast to the 3′ terminal variations, the 5′ terminal variations are predicted to have more impact on the determination of targets [41,59]. This is because in mature miRNAs, the 5′ terminal sequences are used for guiding AGO protein to cleave the targets.

Recent studies have shown that miRNAs can be generated from loop strands and function [42,43]. In line with this new discovery, we identified several sRNAs derived from the loops of the hairpin structures of MIR399b and MIR399e-1 (Additional file 3: Figure S4) that were able to cleave their predicted targets (Additional file 12: Table S11). However, how these functioning loop-derived sRNAs were generated is unknown. It is likely that an alternative miRNA processing pathway may apply, which produces miRNA-like sRNAs independent of the Dicer process. This has been the case for mirtrons [61-63], snoRNA-derived miRNAs [64] and tRNA-derived miRNAs [65]. Another possibility is that pre-miRNAs could directly be loaded into the miRISC and processed into mature miRNAs or functional sRNAs by an unknown exonuclease. Nevertheless, the emerging number of studies in this area suggests that loop-derived sRNAs or miRNAs may be conserved across different species. Future studies are needed to unravel the mechanisms of biogenesis and function of loop-derived sRNAs or miRNAs.

It is commonly understood that only one strand (guide strand) from the miRNA duplex is incorporated into the miRISC and functional. The other strand (passenger strand or miRNA*) is degraded. However, we found that both guide and passenger strands of miR827 were abundant, especially in the P-deficient barley dataset. This raises the possibility that the abundant passenger strand of miR827 may also be incorporated into the miRISC and hence stabilized. If this is true, then it is expected that the passenger strand of miR827 can function as a guide strand. Indeed, we detected cleavage products of the passenger strand of miR827 in the barley degradome library. Our result fits recent data from animal systems, showing that passenger strands are incorporated into the miRISC and functional [40,66-68]. The passenger strand of miR827 may also be able to inhibit target translation. This is because we found no cleavage product in the degradome library for a predicted target (gb|GAKM01126847.1|) which contains a site perfectly reverse complementary to the passenger strand of miR827 at the 3′ untranslational region. Of course, we cannot exclude the possibility that the predicted target may not be a bona fide target. Further experiments are required to reveal a protein level of the target.

One of the predicted targets of miR827, which encodes cytochrome P450-like protein (TC275184/MLOC_73301.3), was observed to have an increasing transcription level in P-deficient barley where the level of miR827 also increased (Figure 5). Several possibilities may explain the parallel increases in miRNA and target expression levels. Firstly, cytochrome P450-like TBP may not be a bona fide target of miR827. However, this does not explain why its cleavage product was detected in the barley degradome library. Secondly, the transcription of cytochrome P450-like TBP may be regulated by both miR827 and P status, but that P status is the dominant regulator, thereby resulting in a high expression level of this RNA in P-deficient barley. Thirdly, the cytochrome P450-like TBP may, like *HvPHO2*, be protected by an unknown mimic sequence which interacts with miR827, inhibiting effective activity of the miRNA. The detection of the target cleavage product may result from incomplete protection by the mimic sequence. Finally, the mode of action of miR827 may be through inhibition of translation of the cytochrome P450-like TBP rather than cleavage of the mRNA. This has been reported for other miRNAs. Translation inhibition may occur via prevention of the ribosome from reading the cap or proteolysis after translation of the polypeptide chain [69]. The regulation of miR827 and its target genes may be complex. Similarly, in rice miR827 is co-expressed with its targets, OsSPX-MFS1 and OsSPX-MFS2, in the same cells in response to P deficiency, where OsSPX-MFS1 is down-regulated and OsSPX-MFS2 is up-regulated in the plants [28].

In *Arabidopsis*, miR399 targets *PHO2*, which negatively regulates Pi uptake and root-to-shoot allocation [9]. In barley we found that with increasing levels of miR399, the transcript levels of *HvPHO2* decreased under P deficiency, suggesting that miR399 suppresses the expression of *HvPHO2*. This is consistent with some, but not all, studies of the relationship between miR399 and *PHO2*[9]. However, it is not clear why the highly expressed miR399 target mimic, *HvISP1,* in the same cells could not protect *HvPHO2* from miR399-mediated cleavage. In *Arabidopsis*, over-expression of *IPS1* has been shown to increase accumulation of mRNA of the miR399 target *PHO2*[25]. Other factors, such as *HvPHO2*-associated sRNAs in barley may be involved in the degradation of *HvPHO2.* In support of this suggestion, we found that some sRNA species derived from *HvPHO2* were only present in the P-deficient barley dataset, but not in the P-sufficient dataset (Additional file 3: Figure S5). Furthermore, these sRNAs were all localised to or near the target sites of *HvPHO2* (Additional file 3: Figure S5). We also found that a lot of antisense sRNAs (siRNAs) to *HvPHO2* were only present in the P-deficient barley dataset (Additional file 3: Figure S5). The presence of *HvPHO2*-associated sRNAs in P-deficient barley could substitute for miR399 in mediating the cleavage of *HvPHO2.* This can explain why the expression level of *HvPHO2* was reduced in the presence of high levels of *HvIPS1* transcript. Because *HvPHO2* contains five predicted target sites, these sRNAs may be triggered by miR399 [45,70,71]. However, it cannot be ruled out that part of the sRNA population may be derived from other genes that contain the same sequence as the target sites. Several such genes have been found in the barley EST databases (data not shown).

Previous studies showed that cleavage events triggered by miR399 occurred at target sites 2–5 in *PHO2*[21,45]. In one study, the cleavage sites were predominantly at target sites 2 and 3 [21]. In another study, target sites 3, 4, and 5 were predominant [45]. However, our investigation of a barley degradome library only revealed cleavage sites at target sites 2 and 5, and occurring at positions 10–11 relative to the 5′ end of the miRNA. The cause of discrepancy between previous studies was proposed to be due to relative abundance differences of different miR399 members in the samples [45]. However, because almost all miR399 genes have the same sequence in the first 10 nt that are the key for miRNA function, it is difficult to judge which miRNA is responsible for cleavage at each target site, especially at positions 10–11 relative to the 5′ end of the miRNA. On the other hand, it is unlikely that two miRNA genes would cleave the same target site with equal efficiency. Therefore, we presume that one cleavage site may only be cleaved by one miRNA at a certain position and a certain time, and that both sequence specificity (including mismatch positions) and abundance are important for site cleavage. Why has it been observed in previous studies [21,45] that one target site is cleaved at several positions? One possibility is that the target site could be cleaved by different members of the miR399 family at different times, and another possibility is that some cleavage products may result from natural degradation. The third possibility is that the target site may be cleaved by siRNAs rather miRNAs, as discussed above. Why target site 1, which is perfectly matched to the abundant hvu-miR399, hvu-miR399c and hvu-miR399e, is not cleaved by these or any other miR399 members in this and previous studies [21,45], is not understood. It suggests that cleavage may be a complex process which relies not only on sequence specificity and abundance, but also on the nature of miRNAs and/or other factors such as plant species, tissue specificity, developmental stage and/or environmental conditions.

miR399 and miR827 are not only responsive to low P, but also responsive to low nitrogen (N) in plants [72-74]. Like P, N is also essential in plant growth and development. In Arabidopsis and maize, miR399 and miR827 are down-regulated under low N conditions [73,74]. However, in soybean, the two miRNAs were not detected [75] although as many as 452 other miRNAs have been detected and many of them are responsive to low N stress in this plant [75]. Under other nutrient stresses such as sulfate starvation and high levels of Fe, Cu, Zn, Al, Cd and Hg in soils, the latter of which inhibit plant growth and cause toxicity to plants [76], miR399 and miR827 were not found to be regulated. It is unclear how miR399 and miR827 are responsive to low N in plants. It is likely that low N affects the expression of transcription factors or co-factors that control the transcription of MIR399 and MIR827 genes, thereby reducing the expression of the mature miRNAs [77]. This might be true because previous studies have identified multiple cis-elements (GNATATNC), which are bound by a MYB transcription factor, PHOSPHATE STARVATION RESPONSE 1 (PHR1), in the upstream sequences of all six miR399 genes in Arabidopsis [8,78]. On the other hand, the expression of miR399 and miR827 may also be determined by other factors. In support, miR399 as well as many other nutrient-responsive miRNAs were detected in the phloem of nutrient stressed plants [79-82]. Because the phloem plays a key role in long-distance signalling for many developmental and environmental responses [83], this suggests that miRNAs can be remotely regulated by signals rather have to be regulated physically. From this point of view, miRNAs themselves could function as a signal in systemic regulation of nutrient stress response [78]. This in turn suggests that the stress-regulated miRNAs such as miR399 and miR827 may share a common mechanism in both expression and function. However, this hypothesis needs to be validated via experimental studies.

## Conclusions

This study characterises two most P-regulated miR399 and miR827 in barley and provides their potential functional mechanisms in the relationship with P and the adaptive responses to P deficiency. The results could be used for miR399- or miR827-based engineering in the future for improvement of crop yields with limited P fertilizer.

## Methods

### Prediction and expression profiling

Pallas barley was grown in soil supplied with either 22.5 mg (deficient P) or 75 mg (sufficient P) Pi (KH2PO4) kg-1 dry soil. Basal nutrients and calcium carbonate were added into the soil as previously described [84]. Plant growth conditions were the same as those described [84]. Small RNAs were isolated from the plant shoots that were harvested 16 days after seed imbibitions and sequenced using the 36-base Illumina platform as previously described [30]. Prediction of novel MIR399 and MIR827 genes was performed with a modified miRanalyzer algorithm [31]. Briefly, we mapped all reads to the barley genome (Morex assembly) allowing no mismatches. Reads that map to nearly identical positions in the genome were clustered into “read clusters” in the following way: (i) the reads were sorted by read count (read frequency); (ii) the most frequent read was assigned to the first read cluster (the coordinates of the read cluster are given by the coordinates of the most frequent read); (iii) for all other reads, we checked if the read lay within a window defined by ClusterStart – 3 nt and ClusterEnd + 5 nt on the same strand (flankings were added in order to assign all isomiRs to the same read cluster); (iv) if the read belonged to an existing cluster, the associated read information (sequence and the read count) was added to the cluster; and (v) if the read did not belong to an existing cluster, a new cluster was opened. After clustering all reads, we extracted pairs of read clusters with distances of less than 150 nt between each other, because for bona fide miRNAs there should be two read clusters corresponding to the two arms processed from the pre-miRNA sequence. Next, the genomic sequence spanned by the two read clusters was extracted and the secondary structure and alignment pattern of the derived pre-miRNA was analysed. We retained only those candidates for which: (i) the reads mapped to the stem of the pre-miRNA; (ii) the reads showed little fluctuation around the 5′ start position in the alignment; and (iii) the read representing the 3p arm mapped to a known miRBase miR399 or miR827 sequence, allowing no more than 2 mismatches.

The obtained mature miRNA sequences (the guide strand which is 3p for both miR399 and miR827) were then mapped again with no mismatches to the genome, in order to obtain all genes giving rise to the same mature miRNA. For all mappings the putative pre-miRNA sequence was extracted. We accepted the candidate if a hairpin structure with MFE < = −35 kcal/mol existed and the 3p reads were located on the stem of the hairpin structure, which was predicted using Mfold [85].

To profile the expression values of a given mature miRNA, we first mapped all reads to the barley genome for obtaining the corresponding genomic coordinates for each read, then by means of the coordinates of the mature miR399 and miR827 sequences, we extracted all reads that lay within a window of: start position of the canonical mature miRNA – 3 nt and end position of the canonical mature miRNA + 5 nt. We allowed a window of [−3;+5] around the canonical miRNA in order to detect all isomiRs and to assign them correctly to each miRNA.

### Target genes of miR399 and miR827

Target genes of miR399 and miR827 were first predicted using psRNATarget [86], a plant sRNA target analysis server [87], and were then validated using a barley (cv. Pallas) degradome library constructed using RLM-5'-RACE technology according to Addo-Quaye et al. [88]. Briefly, poly(A) RNA was extracted from total RNA of barley plants using the Oligotex kit (Qiagen) and then ligated with a 5′ RNA adaptor containing a *Mme*I restriction site using T4 RNA ligase (Invitrogen), followed by reverse transcription, second-strand synthesis, *Mm*eI digestion, ligation of a 3′ dsDNA adaptor, gel-purification, and PCR amplification. Amplified PCR products were sequenced with the Illumina HiSeq platform.

### Southern blot hybridization

Southern blot hybridization was carried out according to Shi et al. [89]. Briefly, barley genomic DNA was digested with appropriate restriction enzymes and separated via gel electrophoresis. The DNA was then transferred to a membrane and hybridized with radiolabelled probes. After hybridization, the membrane was washed once at 65°C for 30–45 min in 2xSSC (0.3 M NaCl and 0.03 M sodium citrate) containing 0.1% sodium dodecyl sulphate (SDS), and twice at 65°C for 30–45 min in 1xSSC containing 0.1% SDS. The membrane was subjected to autoradiography at −80°C as required until sufficient probe signal became visible.

### RT-PCR and qRT-PCR

RT-PCR was performed as described [90], and qRT-PCR was conducted using the NCode™ VILO™ miRNA cDNA Synthesis Kit (Invitrogen). Specifically, following isolation of total RNA, all miRNAs and pre-miRNAs in the sample were polyadenlyated and reverse-transcribed using poly A polymerase, ATP, SuperScript™ III RT, and a specially designed universal RT primer in a single reaction. The synthesized first-strand cDNA was then amplified with a forward primer specific to the miRNA of interest and a universal reverse primer provided in the kit. The amplification was performed in an RG 6000 Rotor-Gene real-time thermal cycler (Corbett Research) using the following conditions: 3 minutes at 95°C followed by 45 cycles of 1 s at 95°C, 1 s at 55°C, 30 s at 72°C (fluorescence reading acquired), and 15 s at 81°C. The transcript levels of genes encoding glyceraldehyde 3-Pi dehydrogenase, heat shock protein 70, cyclophilin, and α-tubulin were used as controls [91]. Normalization was carried out using multiple control genes as described by Burton et al. [91].

### Availability of supporting data

All the supporting data are included as additional files.

## Competing interest

The authors declare that they have no competing interests.

## Authors’ contributions

BS and PL conceived the study. BS designed and conducted the study, analysed and interpreted the data, and wrote the manuscript with the assistance of MH, PG and PL. MH analysed and interpreted the data. All the authors read and approved the final manuscript.

## Supplementary Material

Additional file 1: Table S1 Mature miR399 sequences identified in sRNA datasets previously obtained from P-deficient and P-sufficient barley (cv. Pallas, Hackenberg et al. 2012). Click here for file

Additional file 2: Table S2 Sequences of hvu-miR399-3p, hvu-miR399-5p and hvu-MIR399 genes and their locations in the barley genome. Click here for file

Additional file 3: Figure S1 Stem-loop structures of pre-miR399s and pre-miR827. **Figure S2.** Alignment of sRNAs from the barley datasets on hvu-MIR399-1. The sequences marked in colors aligned uniquely to hvu-MIR399-1. For the read in blue we found a putative target site in the degradome data, but this read also aligns perfectly to hvu-MIR399e-1 and hvu-MIR399e-2. **Figure S3.** Stem-loop structure of hvu-MIR827. miR827-3p (green) and miR827-5p (red) sequences. Alternative miR827-3p and miR827-5p sequences (miR827 (alt)) identified from the same hvu-MIR827 are also shown. **Figure S4.** Alignment of sRNAs on hvu-MIR399b and hvu-MIR399e-1. sRNAs mapping to the loop sequences are highlighted in red. **Figure S5.** Alignment of sense and antisense sRNAs from P-deficient and P-sufficient shoots on *HvPHO2, HvIPS1* and *HvIPS2*. Sequences above each of *HvPHO*2 (A), *HvIPS1* (B) and *HvIPS2* (C) are from P-sufficient shoots, while sequences below each of *HvPHO2, HvIPS1* and *HvIPS2* are from P-deficient shoots. Sequences in black are sense sRNAs, while sequences in blue are antisense sRNAs. Read counts of each sRNA are indicated. Click here for file

Additional file 4: Table S3 Sequence alignment of pre-miR399 sequences obtained from miRBase. Click here for file

Additional file 5: Table S4 Expression profile of hvu-miR399-3p and hvu-miR399-5p, determined by analysis of read counts in the barley P-sufficient and P-deficient sequence datasets. Click here for file

Additional file 6: Table S5 Primer sequences used for qRT-PCR of hvu-MIR399 genes. Click here for file

Additional file 7: Table S6 Isomirs of miR399 and miR827 in barley. Click here for file

Additional file 8: Table S7 Predicted targets of miR399 and miR827 in barley, using psRNATarget (Dai and Zhao, 2011). Click here for file

Additional file 9: Table S8 Genes with miR399- and/or miR827-targetted cleavage products detected by RLM-5′-RACE technology from a barley degradome library. Click here for file

Additional file 10: Table S9 Genes with cleavage products targeted by isomirs of miR399 or miR827, detected by RLM-5′-RACE technology from a barley degradome library. Click here for file

Additional file 11: Table S10 Genes with cleavage products targeted by sRNAs derived from the loop structures of miR399b and miR399e, detected by RLM-5′-RACE technology from a barley degradome library. Click here for file

Additional file 12: Table S11 Antisense sequences to pre-miR399 members and pre-miR827, identified from barley small RNA datasets. The abundance of each antisense sequence is given in the table. Click here for file
