# Supplementary material for: Characterization of phosphorus-regulated miR399 and miR827 and their isomirs in barley under phosphorus-sufficient and phosphorus-deficient conditions
Source: BMC Plant Biol. 2013 Dec 13;13:214. doi: 10.1186/1471-2229-13-214 (PMC3878733; doi:10.1186/1471-2229-13-214)
Supplement: Additional file 3: Figure S1 — Stem-loop structures of pre-miR399s and pre-miR827. Figure S2. Alignment of sRNAs from the barley datasets on hvu-MIR399-1. The sequences marked in colors aligned uniquely to hvu-MIR399-1. For the read in blue we found a putative target site in the degradome data, but this read also aligns perfectly to hvu-MIR399e-1 and hvu-MIR399e-2. Figure S3. Stem-loop structure of hvu-MIR827. miR827-3p (green) and miR827-5p (red) sequences. Alternative miR827-3p and miR827-5p sequences (miR827 (alt)) identified from the same hvu-MIR827 are also shown. Figure S4. Alignment of sRNAs on hvu-MIR399b and hvu-MIR399e-1. sRNAs mapping to the loop sequences are highlighted in red. Figure S5. Alignment of sense and antisense sRNAs from P-deficient and P-sufficient shoots on HvPHO2, HvIPS1 and HvIPS2. Sequences above each of HvPHO2 (A), HvIPS1 (B) and HvIPS2 (C) are from P-sufficient shoots, while sequences below each of HvPHO2, HvIPS1 and HvIPS2 are from P-deficient shoots. Sequences in black are sense sRNAs, while sequences in blue are antisense sRNAs. Read counts of each sRNA are indicated. [file 1471-2229-13-214-S3.pdf]

miR399-1 miR399-2 miR399-3 miR399-4 miR399b miR399e-1 miR399e-2 miR399c miR399d (Barke) miR399d (Morex) miR399k hvu-MIR827

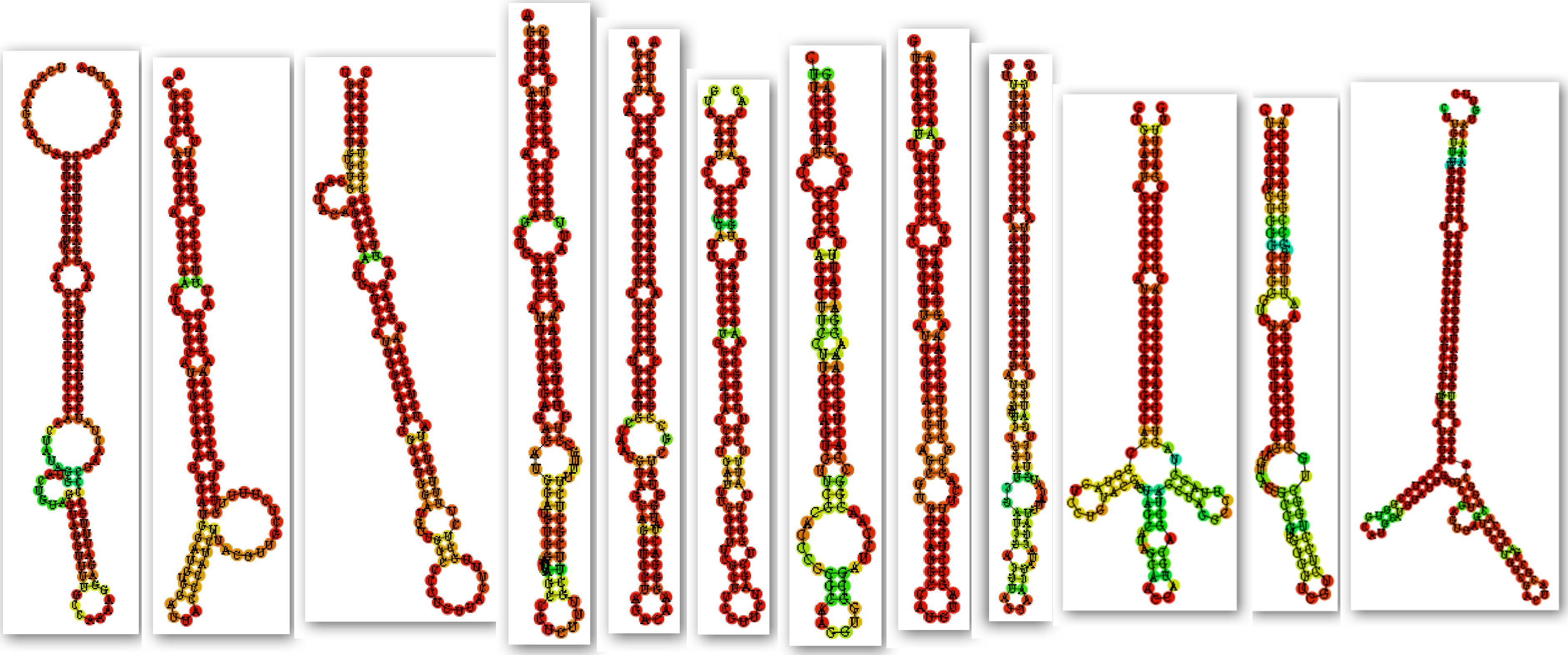

Supplemental Figure 1

hvu-MIR399-1

```
P sufficient                                                                                                                           read count
TCAGAAGAACTAGGTAGATTTGCCAAAGGAGATTTGCCGAACTATAGGTACTGTAGGTAGGTTTTGCCAAAAGGAGATTTGCCCCGAACTATCGGTAGGTTTGCCAAAGGAGATTTGCCCCGAAGAACTTA     6
............(((((((((.((...(((((((((((((......((.......((((((((((........))))))))))))......))))))))))).))...))))))))))).............
                                                                                                     TGCCAAAGGAGATTTGCCCCG              4
                                                                                                     TGCCAAAGGAGAGTTGCCCC               1
                           GGAGATTTGCCGAACTATAGGT                                                                                       1

P deficient
TCAGAAGAACTAGGTAGATTTGCCAAAGGAGATTTGCCGAACTATAGGTACTGTAGGTAGGTTTTGCCAAAAGGAGATTTGCCCCGAACTATCGGTAGGTTTGCCAAAGGAGATTTGCCCCGAAGAACTTA     168 (total count)
............(((((((((.((...(((((((((((((......((.......((((((((((........))))))))))))......))))))))))).))...))))))))))).............
                                                                                                     TGCCAAAGGAGATTTGCCCCG              123
                                                                                                          AAGGAGATTTGCCCCG              4
                                                                                                     TGCCAAAGGAGATTTGC                  4
                                                                      AAGGAGATTTGCCCCG                                                  4
                    TGCCAAAGGAGATTTGC                                                                                                   4
                                          TATAGGTACTGTAGGTAGGTTT                                                                        3
                                        ACTATAGGTACTGTAGGTAGGTT                                                                         3
                                                                                                     TGCCAAAGGAGATTTGCCC                2
                                                                                                     TGCCAAAGGAGATTTGCC                 2
                    TGCCAAAGGAGATTTGCC                                                                                                  2
  AGAAGAACTAGGTAGATTTGC                                                                                                                 2
TCAGAAGAACTAGGTAGATTTG                                                                                                                  2
                    TGCCAAAGGAGATTTGCCCAA                                                                                               2
                                                                                                     TGCCAAAGGAGATTTGCCCCGAA            1
                                                                                                     TGCCAAAGGAGATTTGCCCCGA             1
                                                                                                     TGCCCAAGGAGATTTGCCCCG              1
                                                                                                      GCCAAAGGAGATTTGCCCCG              1
                                                                                                     TGCCAAAGGAGATTTTCCCCG              1
                                                                                                     TGCCAAAGGAGACTTGCCCCG              1
                                                                                                     TGCCAAAGGAGAGTTGCCCC               1
                                                                                                     TGCCAAAGGAGATTTGCCCC               1
                                                                       AGGAGATTTGCCCCGAACTAT                                            1
                                               GTACTGTAGGTAGGTTTTGCCAAA                                                                 1
                                               GTACTGTAGGTAGGTTTTGCCAA                                                                  1
                                               GTACTGTAGGTAGGTTTTGCC                                                                    1
                                       AACTATAGGTACTGTAGGTAGG                                                                           1
```

Supplemental Figure 2

miR827

Read count

```
         TTTTGTTGGTTGTCATCTAACC                        (169)
5'
--- T     T      T                  TT  A    -
   C TGTTT GTTGGT GTCATCTAACCATCATCG  CC TCTG
   | ||||| |||||| ||||||||||||||||||  || ||||
   G ACAAA CGACTA CAGTAGATTGGTAGTAGT  GG AGAC
CTT T     -      C                  --  C    A
3'
     ACAAA-CGACTACCAGTAGATT                            (8935)
```

miR827 (alt)

```
                  TTGTCATCTAACCATCATCG                 (2)
5'
--- T     T      T                  TT  A    -
   C TGTTT GTTGGT GTCATCTAACCATCATCG  CC TCTG
   | ||||| |||||| ||||||||||||||||||  || ||||
   G ACAAA CGACTA CAGTAGATTGGTAGTAGT  GG AGAC
CTT T     -      C                  --  C    A
3'
              CTACCAGTAGATTGGTAGTA                     (10)
```

Supplemental Figure 3

hvu-MIR399b

```
                                                          loop
                                                           V
AGAATCACAGTGCAGTTCTCCTCTGGCATGGATGCCAATGTAGCAGGTCCTAGACAAGGGACTATGGTATCGCCGTCCCTGCCAAAGGAGAATTGCCCTGCCATTCA  324(53.4/30,325.7) adj:324+
.((((..(((.(((((((((((.(((((.(((((.(...(((.((((((((......))))))).)).))).).))))).))))).))))))))))))).))).)..)))))).
                                                                                   TGCCAAAGGAGAATTGCCCTG        140(23.1/13,103.7) adj:140+ hvu-miR399b-3p exact-
         GTGCAGTTCTCCTCTGGCATG                                                                                   133(21.9/12,448.5) adj:133+ hvu-miR399b-5p exact-
                                                                                   TGCCAAAGGAGAATTGCCC          7(1.2/655.2) adj:7+.lv3p|lv3pT|lv3p#-2-
         GTGCAGTTCTCCTCTGGCA                                                                                     4(0.7/374.4) adj:4+.lv3p|lv3pT|lv3p#-2-
         GTGCAGTTCTCCTCTGGCATGA                                                                                  4(0.7/374.4) adj:4+.nta#A|nta#A#1-
                                                                                     CCAAAGGAGAATTGCCCTGCG      4(0.7/374.4) adj:4+.nta#G|nta#G#1-
                                                                                   TGCCAAAGGAGAATTGCC           3(0.5/280.8) adj:3+.lv3p|lv3pT|lv3p#-3-
                                                                                   TGCCAAAGGAGAATTGCCCTGA       3(0.5/280.8)adj:3+.nta#A|nta#A#1-
                                                                                      CAAAGGAGAATTGCCCTG        2(0.3/187.2) adj:2+.lv5p|lv5pT|lv5p#-3-
                                                                                   TGCCAAAGGAGAATTGCCCT         2(0.3/187.2) adj:2+.lv3p|lv3pT|lv3p#-1-
                                                                                   TGCCAAAGGAGAATTGCCCTGAA      2(0.3/187.2) adj:2+.nta#A|nta#A#2-
          TGCAGTTCTCCTCTGGCATG                                                                                   2(0.3/187.2) adj:2+.lv5p|lv5pT|lv5p#-1-
                                                                                    GCCAAAGGAGAATTGCCCTG        2(0.3/187.2) adj:2+.lv5p|lv5pT|lv5p#-1-
                                                     TAGACAAGGGACTATGGTA                                         2(0.3/187.2) adj:2+..
         GTGCAGTTCTCCTCTGGCAT                                                                                    1(0.2/93.6) adj:1+.lv3p|lv3pT|lv3p#-1-
                                                     TAGACAAGGGACTATGGTATCG                                      1(0.2/93.6) adj:1+...
         GTGCAGTTCTCCTCTGGCATC                                                                                   1(0.2/93.6) adj:1+.nta#C|nta#C#1-
                                                                                     CCAAAGGAGAATTGCCCT         1(0.2/93.6) adj:1+.mv-
                                                                                     CCAAAGGAGAATTGCCCTG        1(0.2/93.6) adj:1+.lv5p|lv5pT|lv5p#-2-
                                                                                     CCAAAGGAGAATTGC            1(0.2/93.6) adj:1+.mv-
                                                                                        AAGGAGAATTGCCCTG        1(0.2/93.6) adj:1+.lv5p|lv5pT|lv5p#-5-
                                                                                   TGCCAAAGGAGAATTGCCCTGT       1(0.2/93.6) adj:1+.nta#T|nta#T#1-
                                                     TAGACAAGGGACTATGGTAT                                        1(0.2/93.6) adj:1+...
                                                                                  CTGCCAAAGGAGAATTGCCCTG        1(0.2/93.6) adj:1+.lv5p|lv5pE|lv5p#1-
                                                     TAGACAAGGGACTATGGTATC                                       1(0.2/93.6) adj:1+...
             AGTTCTCCTCTGGCATG                                                                                   1(0.2/93.6) adj:1+. lv5p|lv5pT|lv5p#-4-
                                                                                    GCCAAAGGAGAATTGCCCTGA       1(0.2/93.6) adj:1+.nta#A|nta#A#1-
                                                                                   TGCCAAAGGAGAATTGC            1(0.2/93.6) adj:1+.lv3p|lv3pT|lv3p#-4-
```

hvu-MIR399e-1

```
                                                            loop
                                                           V
GTAGATTACCGGGCCATTTCTCCTTGGGCAGACCGTGATTTGGCTTCGCTTCGTTCTAGCTGGCTTATTTCGTTCTGCCAAAGGAGATTTGCCCAGCAATCCAC  296(48.8/27,705) adj:169.4+
...((((.(.((((.(..(((((((.((((((.((.(((..((((..(((.......))).)))).))).)).)))))).))))))) ..))))).).))))...
                                                                               TGCCAAAGGAGATTTGCCCAG        226(37.2/21,153.1)adj:113+hvu-miR399e-3pexact-
          GGGCCATTTCTCCTTGGGCAGA                                                                            34(5.6/3,182.3) adj:34+hvu-miR399e-5p exact-
                                                                                  CAAAGGAGATTTGCCCAGCAA     4(0.7/374.4)adj:4+.mv-
                                                                               TGCCAAAGGAGATTTGC            4(0.7/374.4)adj:0.6+.lv3p|lv3pT|lv3p#-4-
                                                                                GCCAAAGGAGATTTGCCCAG        3(0.5/280.8)adj:1.5+.lv5p|lv5pT|lv5p#-1-
                                                                             TCTGCCAAAGGAGATTTGCCC          3(0.5/280.8)adj:0.8+.mv-
                                                                                    AAGGAGATTTGCCCAGCAATC   3(0.5/280.8)adj:3+.mv-
                                                                               TGCCAAAGGAGATTTGCCCAGT       2(0.3/187.2)adj:1+.nta#T|nta#T#1-
                                                                               TGCCAAAGGAGATTTGCC           2(0.3/187.2)adj:0.3+.lv3p|lv3pT|lv3p#-3-
                                                CTTCGTTCTAGCTGGC                                            2(0.3/187.2)adj:2+...
                                                                               TGCCAAAGGAGATTTGCCC          2(0.3/187.2)adj:0.3+.lv3p|lv3pT|lv3p#-2-
                                                                               TGCCAAAGGAGATTTGCCCAA        2(0.3/187.2)adj:1+.nta#A|nta#A#1-
                                                                               TGCCAAAGGAGATTTGCCCAGA       1(0.2/93.6) adj:0.5+.nta#A|nta#A#1-
                                                                                  CAAAGGAGATTTGCCCAGCAAT    1(0.2/93.6) adj:1+.mv-
                                                                                    AAGGAGATTTGCCCAGCAAT    1(0.2/93.6) adj:1+.mv-
                                                                                   AAAGGAGATTTGCCCAGCAATC   1(0.2/93.6) adj:1+.mv-
          GGGCCATTTCTCCTTGGGCAGAA                                                                           1(0.2/93.6) adj:1+.nta#A|nta#A#1-
                                                                               TGCCAAAGGAGATTTGCCCAT        1(0.2/93.6) adj:0.5+.nta#T|nta#T#1-
    ATTACCGGGCCATTTCTCCTTGGGC                                                                               1(0.2/93.6) adj:1+...
    ATTACCGGGCCATTTCTCCTTGCGC                                                                               1(0.2/93.6) adj:1+...
                                                                                    AAGGAGATTTGCCCAGCAATT   1(0.2/93.6) adj:1+.nta#T|nta#T#1-
```

Supplemental Figure 4

A

+P (reads above the gene sequence; none shown)

HvPHO2 5'---TTCTGTAAGTTCTT<u>CGGGGCAAATCTCCTTTGGCA</u>---ATAGTT<u>CGGGGCAAATCTCCTTTTGGCA</u>AAACCTACCTACAGTACCTATAG<u>TTCGGCAAATCTCCTTTGGCA</u>AATCTACCTAGTTCTTCTGATCTCCTTCCTTACATATG---TTGCTCCCTGGACAAATCTCCT---TAGGTTTAGCGTCAGTCCTGT<u>CTGGGCGAACCTCCTTTGACA</u>CTAAATCT---<u>CTGGGCAAATCTCCTTTGGCG</u>---3'

-P

Sequencing read	read count
TTCTGTAAGTTCTTCGGGGCA	2
GTACCTATAGTTCGGCAAATCTCC	1
TAGGTTTAGCGTCAGTCCTG	1
AAGCCCCGTTTAGAGGAAACCGT	1
AGCCCCGTTTAGAGGAAACCGT	1
GCCCCGTTTAGAGGAAACCGT	1
GCCCCGTTTAGAGGAAACCG	1
CCCCGTTTAGAGGAAACCGT	112
CCCGTTTAGAGGAAACCGT	6
CCGTTTAGAGGAAACCGT	5
CGTTTAGAGGAAACCGT	3
TATCAAGCCCCGTTTAGAGGA	1
AAACCGTTTTGGATGGATGTCATG	1
AACCGTTTTGGATGGATGTCATG	1
CCGTTTTGGATGGATGTCATG	1
TTTGGATGGATGTCATGGATAT	1
TTGGATGGATGTCATGGATATCA	2
GGATGGATGTCATGGATATCAA	1
CCGTTTAGAGGAAACCGT	203
CGTTTAGAGGAAACCGT	112
CGTTTAGATGGATCAAGAAGA	2
GTTTAGATGGATCAAGAAGACT	1
TAGATGGATCAAGAAGACTAGA	2
AGATGGATCAAGAAGACTAGA	2
ATGGATCAAGAAGACTAGAGGA	1
TGGATCAAGAAGACTAGAGGAA	1
GGATCAAGAAGACTAGAGGAA	2
AGACTAGAGGAAGGAATGTATA	2
AGACTAGAGGAAGGAATGTATAC	1
AACGAGGGACCTGTTTAGAGGA	1
AATCGCAGTCAGGACAGACCCG	1
TCGCAGTCAGGACAGACCCGCT	1
GACCCGCTTGGAGGAAACTGTG	1
TGGAGGAAACTGTGATTTAGA	1
GACCCGTTTAGAGGAAACCG	203

B

+P (reads above the gene sequence; none shown)

HvIPS1 5'---TCGGAGTGTGCCCTCGGCCA---AGGAAGCTCTGGGCGAAACGGCCA---GGCGGCGGCGACTTCTCA---<u>GGGCAACTTCTATCCTTTGGCA</u>---ATGTGTATTGTATCCTAAA---ATGGGTGTATGTGTGATCCTGTT---3'

-P

Sequencing read	read count
TCGGAGTGTGCCCTCGGCCA	1
AGGAAGCTCTGGGCGAAACGGCCA	1
GGCGGCGGCGACTTCTCA	1
ATGTGTATTGTATCCTAAA	1

C

+P

Sequencing read	read count
AGCTAGCTAGCTAGGCGGGTG	1
CGTGTACGTAGCTCCTGTCGGC	1

HvIPS2 5'---ACGCCAGCCACTGCTAGCTAGCTGGCGTGTA**CGTAGCTCCTGTCGGCGA**CGACCGGCCGGCGA---AGCTAGCTAGCTAGGCGGGTG---<u>GGGCAACTATACATCTTTGGCA</u>ATGTCTTCTTCTTCTTCCTCCTCCTCCGATGGATTATTGGAAGATTGTA---GTAAACTGTGTCCGCATCCTT---3'

-P

Sequencing read	read count
GCTAGCTGGCGTGTACGTAGCTCCT	1
CGTGTACGTAGCTCCTGTCGGC	1
CGTAGCTCCTGTCGGCGA	1
AGCTAGCTAGCTAGGCGGGTG	1
GGGCAACTATACATCTTTGGCA	1
TGGATTATTGGAAGATTGTA	1
TGCGGTCGGTGACGATCGATC	2
CCGCACATGCATCGAGGACAGC	1
CGCACATGCATCGAGGACAGC	1
CAGCCGCTGCTGGCCGGCCGCT	1
AGAAGAAGAAGAAGGAGGAGGAG	1
CATTTGACACAGGCGTAGGAA	1

Note:

1. Sequencing reads in black are identical to the gene sequence.
2. Sequencing reads in blue are reverse complementary to the gene sequence.
3. miR399 target sites and target mimicking sequences are underlined.
4. Sequencing reads above the gene sequence were from P deficient (-P) barley shoots, while sequencing reads below the gene sequence were from P sufficient (+P) barley shoots.

Supplemental Figure 5
